# Intimate partner violence and its relation to sexual health outcomes across different adult populations: a systematic review

**DOI:** 10.3389/fsoc.2024.1498969

**Published:** 2024-12-13

**Authors:** Cristobal Calvillo, Alexandra Marshall, Stefani Gafford, Brooke E. E. Montgomery

**Affiliations:** ^1^Department of Health Behavior and Health Education, University of Arkansas for Medical Sciences, Little Rock, AR, United States; ^2^Education and Research Services, University of Arkansas for Medical Sciences, Little Rock, AR, United States

**Keywords:** intimate partner violence, sexual health, systematic review, gender, sexual orientation

## Abstract

**Introduction:**

Intimate Partner Violence (IPV) significantly impacts adults' wellbeing, causing both physical and psychological harm. IPV has been consistently linked to adverse sexual health outcomes, including an increased risk of sexually transmitted infections, unintended pregnancies, and sexual dysfunction. This systematic review examines the evolving relationship between IPV and sexual health outcomes in adults from 2014 to 2024, addressing gaps in understanding across diverse populations and exploring the complex interplay between violence, sexuality, and health.

**Methods:**

A comprehensive search of multiple databases was conducted for peer-reviewed articles published between January 2014 and February 2024. Studies examining the association between IPV and sexual health variables in adult populations (aged 18 and older) were included. The review followed PRISMA guidelines, and 27 articles met the inclusion criteria after full-text screening and quality assessment.

**Results:**

IPV was consistently associated with poorer sexual health outcomes across diverse populations and contexts. Studies utilized various validated instruments to assess IPV and sexual health. Research primarily focused on cisgender heterosexual women, with limited studies on cisgender heterosexual men, men who have sex with men, and transgender individuals. IPV was linked to an increased risk of sexually transmitted infections, unintended pregnancies, sexual dysfunction, and decreased sexual satisfaction. The relationship between IPV and sexual health was influenced by factors such as gender identity, sexual orientation, and cultural context.

**Discussion:**

The review highlights the complex relationship between IPV and sexual health, emphasizing the need for targeted interventions and culturally sensitive approaches. Significant research gaps exist, particularly regarding lesbian women and non-binary individuals. Future studies should employ mixed-methods approaches and consider intersectionality to provide a more comprehensive understanding of IPV's impact on sexual health across diverse populations.

## 1 Introduction

Intimate Partner Violence (IPV) significantly impacts the wellbeing of adults by causing physical, psychological, and long-lasting effects (Barnes et al., 2022; Jordan, 2023; Karakurt et al., 2022; Olive et al., 2021). Additionally, the chronic and complex nature of IPV's physical and psychological impacts underscores the need for interventions focused on enhancing mental health, safety, and support (Wright et al., 2021). In terms of gender, IPV is widely recognized as a major cause of poor health, disability, and mortality among women (Coker, 2007). IPV refers to abusive behavior within an intimate relationship, causing physical, sexual, or psychological harm [(Center for Disease Control Prevention, 2024a; World Health Organization, 2022)], and can occur between current or former romantic partners and is a significant public health issue (Center for Disease Control Prevention, 2024b), impacting lifelong health and wellbeing.

### 1.1 IPV in adult romantic relationships

Research on IPV and its impact on adults has been a focus of recent studies. For example, Semenza et al. (2024) examined the relationship between physical IPV victimization and emotional wellbeing, specifically threat sensitivity, intolerance of uncertainty, and impulse control, as well as access to resources for emotional regulation. This study highlighted the importance of understanding the emotional harm caused by IPV in romantic relationships. In this same line, the experiences of young women with IPV within romantic relationships have also been studied, focusing on the impact on education and overall wellbeing (Klencakova et al., 2023). In addition, in a recent systematic review and meta-analysis, White et al. (2024) showed that IPV is highly prevalent among women and is associated with increased odds of mental health like depression, post-traumatic stress disorder (PTSD), and suicidality. Overall, research indicates that IPV can have significant negative outcomes. Understanding the various outcomes and domains affected by IPV is crucial for developing interventions and support systems for individuals experiencing IPV within romantic relationships.

### 1.2 IPV and sexual health

Research on IPV and its impact on sexual health has been a significant area of study in recent years worldwide. To build on this knowledge, sexual health is defined as “a state of physical, emotional, mental, and social wellbeing in relation to sexuality” (World Health Organization, 2017, p. 3). This definition highlights the importance of promoting safe and satisfying sexual experiences that are free from violence, discrimination, and coercion (World Health Organization, 2017). Moreover, sexual health is connected to Human and Sexual Rights, enabling individuals to enjoy and control their sexual behavior without fear or stigma, which in turn supports healthy sexual relationships (Aggleton et al., 2014; World Health Organization, 2017). Additionally, it has implications for public health outcomes, encompassing sexually transmitted infections (STIs) concerns and issues surrounding abortion, both of which are exacerbated by intimate partner violence. IPV has shown a significant negative impact on sexual health in adults, affecting both men and women. A study carried out in Spain showed that Spanish men and women who experienced physical or non-physical abuse within intimate relationships exhibited poorer sexual health outcomes, including sexual desire, sexual arousal, erection, ability to orgasm, and sexual satisfaction (Sierra et al., 2021). In other studies, sexual violence, a prevalent aspect of IPV, has been associated with detrimental consequences for women's psychological sexual wellbeing, highlighting the profound negative effects on women's health and quality of life (Barbara et al., 2022; Jordan, 2023). In the systematic review by Coker (2007), 51 quantitative studies from 1966 to 2006 consistently showed associations between physical IPV and sexual health issues, including STIs and risky sexual behaviors. All these findings underscore the urgent need for a comprehensive and multidisciplinary approach, involving professionals from various fields such as healthcare providers, psychologists, and sexologists, to address the implications of IPV for sexual health and provide effective support to survivors (Barbara et al., 2022; Wright et al., 2021). Furthermore, to promote overall sexual wellbeing, researchers and healthcare professionals must recognize and address the intricate relationship between IPV and sexual health so they can develop more effective strategies to support adults in achieving optimal sexual health and overall wellbeing.

A recent systematic review of the relationship between IPV and sexual health could yield a more robust and up-to-date understanding of this issue. Previous studies, including work Coker's (2007), have highlighted the need for further research on IPV's impact on sexual health. Besides, such a review could highlight areas where current research is limited and provide valuable insights for new approaches to IPV adults in romantic relationships. Therefore, this study aims to address key gaps by reviewing recent literature (2014–2024) on the negative effects of IPV on adult sexual health. It will examine IPV's impact on sexual health indicators, explore mechanisms across genders, and provide insights to guide future research and interventions. The selected 10-year period is appropriate for gathering a substantial number of relevant studies and identifying trends in the literature. The decision to use 2014 as a starting point is justified, as 17 countries had legalized same-sex marriage by that year, with an additional 18 countries doing so between 2014 and 2024. This increase in the visibility of same-sex relationships is likely to promote greater attention to issues such as IPV within same-sex marriages. The analyzed studies are expected to reflect this heightened visibility and its implications for sexual health. The cutoff date of 2024 ensures that the review incorporates the most current literature, taking into account significant changes in relational dynamics in this context.

The review questions were:

A. How has research on the relationship between IPV and sexual health evolved from 2014 to 2024?
B. What have been the most common assessment tools used to evaluate IPV and sexual health variables in the reviewed studies?
C. What have been the key findings regarding the relationship between different types of IPV and sexual health outcomes across different populations?

## 2 Method

### 2.1 Protocol

The protocol for this systematic review adhered to the guidelines set forth by the Preferred Reporting Items for Systematic Reviews and Meta-Analyses (PRISMA) (Page et al., 2021).

### 2.2 Search strategy

A systematic search of databases was conducted by the medical librarian of the University of [blinded] in February 2024. Databases searched included PubMed MEDLINE, Embase, Web of Science, PsychINFO, PsychArticles, SocINDEX, Journals@OVID, and ProQuest Central. Databases were searched for peer-reviewed articles published between January 2014 and February 2024 and a combination of keywords and controlled vocabulary were used where appropriate. A Boolean search syntax using the operators “AND” and “OR” was applied. The keywords “Intimate Partner Violence”, “Intimate Partner Violence”, “Intimate Partner Abuse”, “Dating Violence”, and “Spouse abuse” were utilized for IPV-related construct. The keywords “Sexual Health”, “Sexual Dysfunctions, Psychological”, “Sexual Dysfunction, Physiological”, “Libido”, “Erectile Dysfunction”, “Orgasm”, “Sexual Arousal”, “Sexual health”, “Sexual dysfunction”, “Psychosexual dysfunction”, “Psychosexual disorder”, “Sexual pleasure”, “Sexual desire”, “Hypoactive sexual desire disorder”, “Sexual aversion disorder”, “Sexual arousal”, “Sexual arousal disorder”, “Erectile dysfunction”, “Orgasm”, “Orgasmic disorder”, “Anorgasmia”, “Sexual satisfaction”, and “Sexual gratification” were utilized for any sexual health construct. An example of PubMed search is as follows {[“Intimate Partner Violence”[Mesh] OR “Intimate Partner Violence”[tiab] OR “Intimate Partner Abuse”[tiab] OR “Dating Violence”[tiab] OR “Spouse abuse”[tiab]] AND [“Sexual Health”[Mesh] OR “Sexual Dysfunctions, Psychological”[Mesh] OR “Sexual Dysfunction, Physiological”[Mesh] OR “Libido”[Mesh] OR “Erectile Dysfunction”[Mesh] OR “Orgasm”[Mesh] OR “Sexual Arousal”[Mesh] OR “Sexual health”[tiab] OR “Sexual dysfunction”[tiab] OR “Psychosexual dysfunction”[tiab] OR “Psychosexual disorder”[tiab] OR “Sexual pleasure”[tiab] OR “Sexual desire”[tiab] OR “Hypoactive sexual desire disorder”[tiab] OR “Sexual aversion disorder”[tiab] OR “Sexual arousal”[tiab] OR “Sexual arousal disorder”[tiab] OR “Erectile dysfunction”[tiab] OR “Orgasm”[tiab] OR “Orgasmic disorder”[tiab] OR “Anorgasmia”[tiab] OR “Sexual satisfaction”[tiab] OR “Sexual gratification”[tiab]]}.

### 2.3 Eligibility criteria

The eligibility criteria for this systematic review encompassed the following: (a) Inclusion of studies examining intimate partner violence (IPV) alongside variables related to sexual health; (b) inclusion of studies establishing an association between IPV and sexual health variables; (c) consideration of studies employing quantitative, qualitative, or mixed-methods research designs, particularly those of a descriptive-empirical nature; (d) inclusion of studies involving adult participants; and, (e) acceptance of articles published in either English or Spanish. Conversely, exclusion criteria encompassed documents employing alternative methodological designs (such as systematic or bibliographic reviews, or meta-analysis), different document types (e.g., doctoral theses or other academic works), studies conducted in languages other than English or Spanish, studies featuring adolescents and/or children as participants, and studies lacking an analysis of both sexual health and their relationship with IPV variables.

### 2.4 Study selection

Initially, 314 article citations were retrieved, resulting in 459 citations after deduplication using EndNote bibliographic software and Covidence systematic review software. Two hundred and two citations were screened by title and abstract, and 53 articles proceeded to full-text screening. Covidence systematic review software was utilized to screen each retrieved record and assess its alignment with the inclusion criteria specified for this review.

### 2.5 Data extraction and quality assessment

The risk of bias in study selection was evaluated by independently assessing the scientific quality, robustness, methodological transparency, and reliability of results using two different assessment tools. One tool was based on the guidelines outlined by Loney et al. (1998), and the other was the Evidence Appraisal of a Single Study Form for Descriptive Study, Epidemiology Study, and Case Series provided by LEGEND Evidence Evaluation Tools and Resources (Cincinnati Children's Hospital Medical Center, 2024). These tools facilitated the evaluation of aspects such as the appropriateness of the study method and the unbiased measurement of outcomes. The two assessment tools for this study were also adapted to evaluate qualitative studies, with questions tailored accordingly. After full-text screening and assessment, 27 articles were included in the review. The list of the 27 included papers is shown in Table 1. Since no human participation was involved, ethical review requirements were not applicable. For this work, it was tabulated, for better visualization, the authors and year of publication, the location where the study was carried out, the samples, type of samples, type of study's methodology, measure(s) used for assessing IPV and sexual health variables, key finding related to the association of interest.

**Table 1:** Information about included studies.

**Authors and year**	**Location**	**Sample**	**Type of sample**				**Type of the study's methodology (Qt or Ql)**	**Form of IPV evaluation**	**Form of any sexual health construct evaluation**	**Key findings related to the association of interest**
			**Clinical**	**Student**	**General**	**Other**				
Bagwell-Gray (2019)	USA	Twenty-eight women from a metropolitan area in the southwestern USA, including survivors seeking services from a domestic violence agency and those not seeking services, recruited through advocacy groups and community outreach.	X				Ql	In-depth interviews focusing on physical, sexual, and psychological abuse, as well as stalking by an intimate partner, including past and present IPV experiences and dynamics of abusive relationships.	In-depth interviews addressing sexual health experiences before, during, and after IPV, perspectives on sexuality, sexual wellbeing, and strategies to maintain or improve sexual health.	IPV was linked to unprotected sex with abusive partners, leading to STI risks, endometriosis, unintended pregnancies, and miscarriages.
Bahrami_Vazir et al. (2020)	Iran	346 partnered primigravida Iranian women who visited health centers for routine pregnancy.	X				Qt	The Revised Conflict Tactics Scales (CTS2: Behboodi-Moghadam et al., 2010; Straus et al., 1996), assessing negotiation, psychological aggression, physical assault, sexual coercion, and injuries during the past year.	The Iranian version of the Female Sexual Functioning Index (FSFI: Mohammadi et al., 2008; Rosen et al., 2000), which evaluates sexual desire, arousal, lubrication, orgasm, satisfaction and pain.	IPV, including psychological aggression, physical assault, sexual coercion, and injury, was associated with a higher likelihood of sexual dysfunction.
Bustos and Pérez (2018)	Chile	305 Chilean women attending gynecological or family planning checkups.	X				Qt	A survey assessing four domains of IPV: psychological, physical, sexual, and economic violence.	Chilean version of the Female Sexual Functioning Index (FSFI: Blümel et al., 2009; Rosen et al., 2000), which evaluates sexual desire, arousal, lubrication, orgasm, satisfaction, and pain.	Psychological IPV, whether mild, moderate, or severe, was significantly associated with poorer sexual functioning.
Callands et al. (2024)	USA	349 sub-Saharan African Liberian participants (170 women and 179 men).			X		Qt	Adapted questions from the Conflict Tactics Scale (CTS: Straus et al., 1996; Vega and O'Leary, 2007), assessing emotional, physical, and sexual violence in current relationship from victimization and perpetration perspective.	Adapted questions from the Liberia Demographic and Health Survey (Liberia Institute of Statistics Geo-Information Services et al., 2014), assessing recent STI diagnosis and symptoms through gender-specific questions.	IPV was associated with STI diagnosis or symptoms in both men and women.
Cha et al. (2023)	USA	7,777 heterosexually active individuals with low socioeconomic status from 17 US urban cities, obtained from the CDC's National HIV Behavioral Surveillance.			X		Qt	*Ad-hoc* questions regarding physical and sexual IPV victimization over the past 12 months.	*Ad-hoc* questions assessing HIV screening, behavioral risk factors for HIV, and STI diagnosis within the past 12 months.	IPV was associated with more frequent HIV screenings, exchanging sex for money or drugs, and condomless sex with HIV-positive partners.
Ferlatte et al. (2018)	Canada	7,872 queer men (64.3% identifying as gay, 21.2% as bisexual, and 14.5% as other).			X		Qt	*Ad-hoc* questions about emotional, physical, and sexual abuse by a partner within the past 12 months.	*Ad-hoc* questions regarding sexual behaviors, including condomless anal intercourse and the number of sexual partners in the past 12 months.	IPV was associated with syphilis diagnoses among participants.
FitzPatrick et al. (2022)	Australia	1,346 pregnant women recruited from six public hospitals in Melbourne, representative of nulliparous women across the state.	X				Qt	Composite Abuse Scale (CAS: Hegarty et al., 2005), assessing physical and emotional abuse within intimate relationships.	Questions drawn from a developed measure (Barrett et al., 2000), assessing sexual health, including resumption of vaginal sex and pain during sex after childbirth.	Emotional and physical IPV were associated with engaging in vaginal sex “too soon” and experiencing painful sex after childbirth.
Gibson et al. (2019)	USA	2,016 midlife and older women from the Kaiser Permanente Northern California healthcare system, most of whom were postmenopausal (82.1%).	X				Qt	Questions from standardized self-administered questionnaires, adapted from past epidemiologic research (Mouton et al., 2010), assessing emotional and physical IPV and sexual assault.	Questions from structured-item questionnaires (Gellis et al., 2010), assessing menopause symptoms, such as vaginal dryness, irritation, and pain with intercourse.	Sexual IPV was associated with vaginal dryness, irritation, and pain with intercourse.
Hellemans et al. (2015)	Belgium	392 participants (197 women and 195 men) of Turkish descent residing in Flanders, Belgium.			X		Qt	Conflict Tactics Scale (CTS: Straus, 1979), with adapted questions to assess physical IPV; modified questions from the WHO Multi-Country Study on Women's Health and Domestic Violence (Garcia-Moreno et al., 2006).	Maudsley Marital Questionnaire (MMQ: Arrindell et al., 1983; Crowe, 1978), assessing sexual satisfaction; Sexual Functioning Scale (SFS: Enzlin et al., 2012), assessing sexual function; *ad-hoc* question about sexual distress; Dyadic Sexual Communication Questionnaire (DSC) (Catania, 1986), assessing sexual communication within the relationship.	Physical and psychological IPV were linked to sexual dysfunction among both women and men, with higher levels of physical IPV increasing the likelihood of sexual dysfunction with distress and psychological IPV raised the odds of sexual dysfunction without distress and with distress.
Hutchinson et al. (2023)	Australia	15,820 Australian women from the Australian Longitudinal Study on Women's Health (ALSWH), covering two age cohorts (1973–78 and 1989–95).			X		Qt	*Ad-hoc* question assessing past experience of a violent relationship with a partner/spouse.	*Ad-hoc* questions assessing sexual orientation, reproductive outcomes (e.g., endometriosis, miscarriage).	IPV was associated with STI diagnoses, endometriosis, infertility, pregnancy termination, and miscarriage, with intergenerational trends and bisexual identity influencing outcomes.
Jiang et al. (2020)	China	976 Chinese men who have sex with men (MSM) living in Guangzhou for over 3 months.			X		Qt	*Ad-hoc* questions assessing experiences of property damage, physical threats, and forced unwanted sex.	*Ad-hoc* questions assessing sexual partner-seeking behavior, condomless anal sex, and child sexual abuse.	IPV was associated with high-risk sexual behaviors, psychological distress affecting sexual decision-making, and barriers to accessing sexual health services.
Kelley et al. (2019)	USA	83,329 postmenopausal women from the Women's Health Initiative clinical centers, recruited between 1993 and 1998.	X				Qt	*Ad-hoc* questions assessing verbal and physical abuse in the previous year.	*Ad-hoc* questions on global sexual satisfaction and sexual frequency satisfaction.	IPV was associated with higher rates of global and frequency-related sexual dissatisfaction.
Loeffen et al. (2016)	The Netherlands	262 women from the Netherlands, with 89.6% being native Dutch.	X				Qt	Composite Abuse Scale (CAS: Hurlbert, 1993), assessing physical and emotional abuse within intimate relationships.	*Ad-hoc* questions regarding gynecological conditions (STIs, vaginal pain, etc.) and pregnancy outcomes (miscarriages, low birth weight, etc.).	IPV was significantly associated with higher prevalence of lower abdominal pain, vaginal discharge, dyspareunia, miscarriage, and low birth weight.
Logie et al. (2016)	Canada	128 women displaced by the 2010 earthquake in Leogane, Haiti, or surrounding areas, living in tent camps or different residences.				X	Qt	*Ad-hoc* question assessing experiences of physical violence by a partner.	Relationship control subscale from the Sexual Relationship Power Scale (SRPS: Pulerwitz et al., 2000), assessing relationship control and condom use.	IPV was associated with lower likelihood of reporting condom use.
Martínez-Madrid et al. (2021)	Spain	187 women divided into premenopausal, perimenopausal, and postmenopausal groups, recruited from healthcare centers.	X				Qt	The Spanish short version of the Woman Abuse Screening Tool (WAST: Brown et al., 1996; Fogarty and Brown, 2002; Plazaola-Castaño et al., 2009), assessing tension and difficulties with an intimate partner as a IPV screening.	The Spanish six-item version of the Female Sexual Functioning Index (FSFI-6: Isidori et al., 2010; Rosen et al., 2000), assessing sexual desire, arousal, lubrication, orgasm, satisfaction and pain.	IPV was negatively associated with sexual functioning across all domains, with lubrication being an exception in postmenopausal women.
Miltz et al. (2019)	England	436 gay men, 40% were born outside the UK.	X				Qt	IPV questions based on the “Health and Relationship Survey” from a previous study in London, assessing psychological, physical, and sexual IPV from victimization and perpetration perspectives.	*Ad-hoc* questions assessing condomless anal sex, number of sexual partners, drug use in sexual contexts; The Internalized Homophobia Scale (IHS: Ross and Rosser, 1996), assessing internalized homophobia in four dimensions: public identification as gay, perception of stigma associated with being homosexual, social comfort with gay men, and the moral and religious acceptability of being gay.	Lifetime and past-year IPV victimization were associated with sexualized drug use and internalized homophobia.
Orchowski et al. (2020)	USA	178 women from a large community college in the Northeast US.		X			Qt	The Conflict Tactics Scale-2 (CTS-2: Straus et al., 1996), assessing physical, psychological, and sexual IPV, negotiation, and injury.	The Sexual Assertiveness Scale (SAS; Morokoff et al., 1997), assessing assertiveness in intimate situations; the Condom Use Resistance Tactics Scale (Davis et al., 2014), assessing women's frequency of engaging in unprotected sex due to condom resistance tactics.	Physical and sexual IPV were associated with frequent unprotected sex due to condom use resistance, while psychological IPV was associated with lower sexual assertiveness.
Overstreet et al. (2015)	USA	186 HIV-negative women from the United States.			X		Qt	Psychological Maltreatment of Women Inventory (PMWI: Tolman, 1989), assessing psychological and physical IPV, as well as sexual coercion within intimate relationships; physical assault subscale from the Conflict Tactics Scale-2 (CTS-2: Straus et al., 2003), assessing physical violence; Sexual Experiences Survey (Koss and Oros, 1982) assessing sexual coercion and forceful sexual experiences in the relationship.	*Ad-hoc* questions assessing sexual risk behaviors over the past 6 months, including unprotected sex with HIV-positive or unknown status partners and engagement in sex trade.	Psychological, physical, and sexual IPV were significantly associated with sexual risk behavior. Psychological IPV was linked to PTSD, which mediated the relationship between IPV and sexual risk behavior.
Parsons et al. (2018)	USA	212 transgender individuals from the New York City metropolitan area, with 59% identifying as female/woman/girl and 52.4% identifying as LGBQ.	X				Qt	The adapted version of the Revised Conflict Tactics Scale (RCTS: Greenwood et al., 2002; Straus et al., 1996), assessing any form of IPV over the past 5 years.	*Ad-hoc* questions, assessing HIV transmission risk events (e.g., condomless sex with a partner of discordant or unknown HIV status) and transactional sex.	IPV was associated with higher rates of transactional sex and HIV transmission risk behaviors.
Peasant et al. (2018)	USA	200 undergraduate American women.		X			Qt	The Hurt-Insult-Threaten-Scream Scale (HITS: Sherin et al., 1998), assessing physical and psychological IPV	Condom Influences Strategy Questionnaire (Noar et al., 2002), assessing six strategies for condom negotiation: Conveying risk information, using deception, using seduction, withholding sex, directly requesting condoms, and relationship conceptualizing.	Psychological IPV was positively associated with lower condom negotiation, leading to decreased condom use with non-casual sex partners.
Porter and Mittal (2022)	USA	173 HIV-negative heterosexual women who have experienced IPV	X				Qt	The Abusive Behavior Inventory (ABI: Zink et al., 2007), assessing psychological, physical, and sexual IPV.	Adaptation of the Self-Efficacy with Steady Partner Scale (Murphy et al., 2001), assessing condom use self-efficacy.	IPV was significantly negatively associated with safer sex self-efficacy.
Rosenthal and Starks (2015)	USA	480 participants (253 women, 224 men, and 3 transgender participants), with various sexual identities (heterosexual, gay/lesbian, bisexual, etc.)				X	Qt	The six-item version of the Conflict Tactics Scale (Agrawal et al., 2009; Straus and Douglas, 2004), assessing emotional, physical, and sexual aggression within the past year.	The 11-item version of the Dyadic Sexual Communication Scale (Catania, 1998; Parsons et al., 2012), assessing sexual communication; the three-item version of the sexual satisfaction subscale of the Derogatis Sexual Functioning Inventory (Derogatis, 1975; Parsons et al., 2012), assessing sexual satisfaction.	IPV in interracial, same-sex, and interracial same-sex couples was significantly negatively associated with sexual satisfaction and communication.
Ruark et al. (2017)	USA	401 participants (89 couples and 223 individuals) from various Malawian ethnic groups.			X		Qt	Questions from the IPV module of the Malawi Demographic and Health Survey (National Statistical Office and ICF Macro, 2011), assessing controlling behavior, emotional, physical, and sexual violence in women, and IPV perpetration in men.	Index of Sexual Satisfaction (ISS: Hudson, 2007), assessing sexual satisfaction.	IPV was significantly associated with lower sexual satisfaction in women, while emotional IPV in men was linked to lower sexual satisfaction.
Sierra et al. (2021)	Spain	3,394 heterosexual individuals (1,628 men and 2,133 women) in relationships.			X		Qt	The Spanish version of Index of Spouse Abuse (Hudson and McIntosh, 1981). The version from Sierra et al. (2011a) was used for women, assessing non-physical abuse and physical abuse. The version of Santos-Iglesias et al. (2013) was used for men, assessing non-physical abuse, behavioral control, and physical abuse.	Spanish versions of multiple scales, including the Sexual Assertiveness Scale (SAS: Morokoff et al., 1997; Sierra et al., 2011b); Sexual Opinion Survey (SOS-6: Fisher et al., 1988; Vallejo-Medina et al., 2014), assessing assertiveness, STI prevention, sexual desire, and sexual functioning; Hurlbert Index of Sexual Fantasy (HISF: Hurlbert, 1993; Sierra et al., 2014), assessing attitudes toward sexual fantasies; Sexual Desire Inventory (SDI: Moyano et al., 2017; Spector et al., 1998), assessing interest in sexual activity; Massachusetts General Hospital Sexual Functioning Questionnaire (MGH-SFQ: Fava et al., 1998; Sierra et al., 2012), assessing sexual functioning.	Physical and non-physical IPV was negatively associated with sexual assertiveness, desire, and functioning in both men and women, with additional effects on STI prevention and sexual fantasies in women.
Storholm et al. (2023)	USA	33 participants, including 23 sexually diverse men who experienced IPV and 10 clinical and social service providers.	X			X	Ql	Semi-structured interviews exploring violence, control, and abuse within intimate relationships.	Semi-structured interviews exploring sexual health behaviors, including condom use, HIV testing, and PrEP use, within the context of IPV.	IPV affected power dynamics in sexual relationships, leading to pressure for condomless sex and varied responses to PrEP use, with some partners encouraging or discouraging its use to prevent aggression.
Tenkorang (2019)	Canada	2,289 ever-married Ghanaian women, representing a cross-section of the general population.			X		Qt	*Ad-hoc* questions assessing physical, sexual, and emotional IPV at the individual and community levels.	*Ad-hoc* questions assessing unwanted pregnancies and pregnancy loss.	IPV was associated with higher prevalence of unwanted pregnancies and pregnancy loss, particularly in communities with high levels of sexual violence.
Willie et al. (2018)	USA	900 women from 49 states and the District of Columbia, stratified by three racial/ethnic groups.			X		Qt	Random digit dial telephone survey assessing experiences of sexual violence, stalking, and IPV among adult women and men.	HIV infection diagnosis rates reported from the Centers for Disease Control and Prevention (CDC).	IPV was positively associated with HIV diagnosis rates, particularly in states with low and moderate IPV protection policies.

## 3 Results

**Figure 1:**
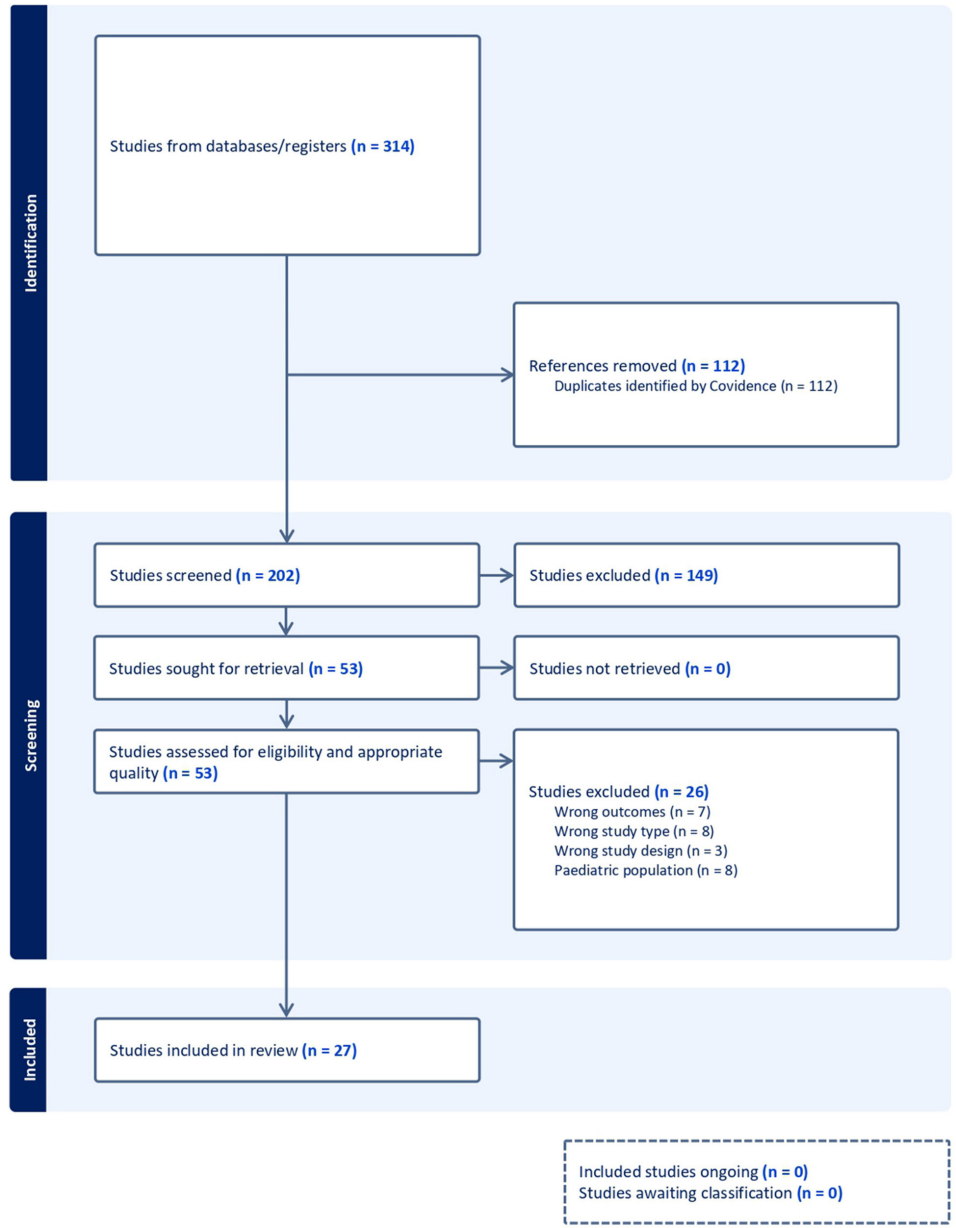
Study selection flow diagram.

As shown in Figure 1, The PRISMA flow chart illustrates the systematic review process for studies on intimate partner violence and sexual health. Initially, 314 records were identified through database searching. After removing duplicates, 202 records were screened. Of these, 149 records were excluded based on title and abstract review. The remaining 53 full-text articles were assessed for eligibility, resulting in 26 studies being excluded. Ultimately, 27 studies were included in the qualitative synthesis for this systematic review. This flow chart provides a clear visual representation of the study selection process, from initial identification to final inclusion, ensuring transparency in the review methodology.

### 3.1 How has research on the relationship between IPV and sexual health evolved from 2014 to 2024?

Our systematic review examined studies investigating the relationship between IPV and sexual health across various contexts. Although the study search spanned from 2014 to 2024, the articles reviewed for this paper that met the inclusion criteria were from 2015 to 2024. As shown in Table 1, notable contributions and sustained interest in this field came from researchers such as Hellemans et al. (2015) or Overstreet et al. (2015) to more recent work by Cha et al. (2023) and Callands et al. (2024). The studies covered a wide geographical range. The research articles reviewed were carried out in countries from the five continents. For example, in Africa, studies were conducted in Ghana (e.g., Tenkorang, 2019), Liberia (e.g., Callands et al., 2024), and Malawi (e.g., Ruark et al., 2017). In the Americas, there was evidence of work in Canada (e.g., Ferlatte et al., 2018), Chile (e.g., Bustos and Pérez, 2018), and the United States (e.g., Cha et al., 2023; Gibson et al., 2019; Kelley et al., 2019). In Asia, research was found in China (e.g., Jiang et al., 2020) and Iran (e.g., Bahrami_Vazir et al., 2020). For Europe, studies were conducted in Belgium (e.g., Hellemans et al., 2015), England (e.g., Miltz et al., 2019), the Netherlands (e.g., Loeffen et al., 2016), and Spain (e.g., Martínez-Madrid et al., 2021; Sierra et al., 2021). Finally, research was also found in Australia (e.g., FitzPatrick et al., 2022; Hutchinson et al., 2023). It is worth mentioning that most of the research was carried out in the United States.

Research on the connection between intimate partner violence (IPV) and sexual health has expanded significantly in recent years, broadening in both scope and geographic reach while incorporating diverse methodologies. Contemporary studies span a variety of global regions and predominantly rely on quantitative approaches, often utilizing standardized IPV assessments. The evidence consistently demonstrates that IPV has a detrimental effect on sexual health, heightening the risk of sexually transmitted infections (STIs), unintended pregnancies, and sexual dissatisfaction. Over the past decade, this body of research has cultivated a deeper, more nuanced understanding of IPV's complex impact on sexual health, revealing negative outcomes not only among heterosexual women but also across a range of different populations.

### 3.2 What have been the most common assessment tools used to evaluate IPV and sexual health variables in the reviewed studies?

Sample sizes varied considerably, ranging from 28 participants in Bagwell-Gray's (2019) qualitative study to 83,329 in Kelley et al.'s (2019) large-scale quantitative research. The majority of studies employed quantitative methodologies (e.g., Hellemans et al., 2015; Jiang et al., 2020), while some used qualitative approaches (e.g., Bagwell-Gray, 2019; Storholm et al., 2023), providing in-depth insights into participants' experiences. There were no mixed-method studies.

#### 3.2.1 Instruments to assess IPV

Quantitative studies commonly employed standardized instruments to assess IPV, such as the Conflict Tactics Scale (CTS; Straus et al., 1996), which includes several subscales measuring various dimensions of IPV, including negotiation, psychological aggression, physical assault, sexual coercion, and injury. Variations of the CTS were used in studies such as Bahrami_Vazir et al. (2020), Callands et al. (2024), Hellemans et al. (2015), Miltz et al. (2019), Orchowski et al. (2020), Overstreet et al. (2015), Parsons et al. (2018), and Rosenthal and Starks (2015), often focusing on specific forms of IPV such as physical violence or psychological aggression. Additionally, the Composite Abuse Scale (CAS; Hegarty et al., 2005) was utilized in studies like FitzPatrick et al. (2022) and Loeffen et al. (2016), which assesses the frequency and severity of both physical and psychological abuse. Other tools included the Abusive Behavior Inventory (ABI; Zink et al., 2007), the Psychological Maltreatment of Women Inventory (PMWI; Tolman, 1989), and the Spanish versions of the Index of Spouse Abuse (Hudson and McIntosh, 1981; Santos-Iglesias et al., 2013; Sierra et al., 2011a), each targeting different aspects of IPV, such as emotional and psychological abuse. The Woman Abuse Screening Tool (WAST; Brown et al., 1996; Fogarty and Brown, 2002; Plazaola-Castaño et al., 2008) was also employed in multiple studies to screen for potential abuse and assess the nature of abusive relationships. Some studies also incorporated *ad-hoc* questions based on established scales (e.g., Cha et al., 2023; Ferlatte et al., 2018), assessing physical, psychological, and/or sexual IPV.

#### 3.2.2 Instruments to assess any sexual health construct

Sexual health dimensions in these studies were evaluated using various instruments. The Female Sexual Function Index (FSFI; Rosen et al., 2000) was used in multiple studies (e.g., Bahrami_Vazir et al., 2020; Bustos and Pérez, 2018; Martínez-Madrid et al., 2021). The Sexual Assertiveness Scale (SAS; Morokoff et al., 1997) and its Spanish version (Sierra et al., 2011b) were utilized in different studies (Orchowski et al., 2020; Sierra et al., 2021). Other instruments used in the same study (Sierra et al., 2021) included the Spanish versions of the Sexual Opinion Survey (SOS-6; Fisher et al., 1988; Vallejo-Medina et al., 2014), the Hurlbert Index of Sexual Fantasy (HISF; Hurlbert, 1993; Sierra et al., 2020), the Sexual Desire Inventory (SDI; Moyano et al., 2017; Spector et al., 1998), and the Massachusetts General Hospital Sexual Functioning Questionnaire (MGH-SFQ; Fava et al., 1998; Sierra et al., 2012). The Dyadic Sexual Communication Questionnaire (DSC; Catania, 1998), the Maudsley Marital Questionnaire (MMQ; Arrindell et al., 1983; Crowe, 1978), and the Sexual Function Scale (SFS; Enzlin et al., 2012) were employed in another study (Hellemans et al., 2015). Additionally, custom questionnaires addressing sexual behaviors and outcomes were used in some studies (e.g., Jiang et al., 2020; Ferlatte et al., 2018).

In contrast, the qualitative studies (e.g., Bagwell-Gray, 2019; Storholm et al., 2023) employed in-depth interviews to explore sexual health experiences before, during, and after IPV, as well as individuals' perspectives on sexuality, sexual wellbeing, and strategies for maintaining or improving sexual health. Also, interviews examined sexual health behaviors, such as condom use, HIV testing, and PrEP use, within the context of IPV. The researcher conducted the interviews with the study participants. By providing rich, contextual data, they shed light on how IPV impacts various aspects of sexual wellbeing, including sexual decision-making, pleasure, and safety.

### 3.3 What have been the key findings regarding the relationship between IPV and sexual health outcomes across different populations?

Across studies, IPV was consistently associated with poorer sexual health outcomes. The relationship between IPV and sexual health proves complex, and heavily influenced by gender identity and sexual orientation. The majority of the studies examined the relationship between IPV and sexual health in cisgender heterosexual women. For example, Overstreet et al. (2015) examined a sample of 186 HIV-negative women in the U.S., finding that psychological, physical, and sexual IPV were significantly associated with risky sexual behaviors, that is, engaging in sexual activities without proper protection. Logie et al. (2016) studied 128 internally displaced women in Haiti after the 2010 earthquake, finding that those who experienced physical violence were less likely to report condom use. Willie et al. (2018) found a significant positive association between state-level IPV rates and HIV diagnosis rates among women in the U.S. In Ghana, Tenkorang (2019) found significant associations between physical and sexual violence and unintended pregnancies among 2,289 married women. Sierra et al. (2021) studied 2,133 heterosexual Spanish women, finding that IPV was associated with reduced assertiveness, STI prevention, sexual fantasies, desire, arousal, orgasm, and satisfaction. Hutchinson et al. (2023) reported that in a sample of 15,820 Australian women, IPV was linked to higher probabilities of STIs, endometriosis, infertility, pregnancy termination, and miscarriage. Physical violence specifically was associated with increased miscarriage risk. In clinical settings, several studies highlighted IPV's impact on women's sexual health. Loeffen et al. (2016) found in a sample of 262 Dutch women that IPV was significantly associated with lower abdominal pain, vaginal discharge, itching/pain, dyspareunia, miscarriage, induced abortion, and low birth weight. Bustos and Pérez (2018) reported that psychological IPV was significantly associated with poorer sexual function among Chilean women attending gynecological or family planning appointments. Gibson et al. (2019) found that among 2,016 middle-aged and older women, sexual IPV was associated with vaginal dryness, irritation, and pain during intercourse. Kelley et al. (2019) reported that in a sample of 83,329 postmenopausal U.S. women, IPV was linked to higher rates of overall and frequency-related sexual dissatisfaction. A qualitative study by Bagwell-Gray (2019) with 28 women found that IPV was related to unprotected sex with an abusive partner, leading to STI risks, endometriosis, unintended pregnancies, and miscarriages. Bahrami_Vazir et al. (2020) found that pregnant Iranian women who experienced IPV were more likely to exhibit sexual dysfunction. Martínez-Madrid et al. (2021) found in a sample of 187 Spanish women that IPV was negatively associated with sexual function in all domains except lubrication in postmenopausal women. Porter and Mittal (2022) reported that among 173 HIV-negative heterosexual women who experienced IPV, there was a significant negative association between IPV and self-efficacy in maintaining safe sexual relationships, specifically in the context of preventing the acquisition of infections. FitzPatrick et al. (2022) found that in a sample of 1,346 pregnant Australian women, emotional and physical IPV both were associated with having vaginal sex “too soon” and painful sex after childbirth. Peasant et al. (2018) reported that among 200 college women, psychological IPV was associated with lower condom use negotiation and, consequently, lower condom use with non-casual partners. Similarly, Orchowski et al. (2020) found that among 178 college women, physical and sexual IPV were associated with frequent unprotected sex due to partner resistance to condom use and lower sexual assertiveness.

Research on IPV and sexual health in cisgender heterosexual men is more limited but provides valuable insights. Ruark et al. (2017) reported that among Malawian men, only emotional IPV was significantly associated with low sexual satisfaction. In the previously mentioned study carried out by Sierra et al. (2021), they also assessed 1,628 heterosexual Spanish men, finding that non-physical and physical abuse both were significantly associated with various negative sexual health outcomes, such as decreased sexual assertiveness, STI prevention, sexual desire, and satisfaction. Hellemans et al. (2015) found in a sample of 195 Turkish men living in Belgium that both physical and psychological IPV were associated with sexual dysfunction. Callands et al. (2024) reported that among 179 sub-Saharan men, IPV experience was associated with STI diagnosis or symptoms. In addition, Cha et al. (2023) examined a larger, more diverse sample of 7,777 low socioeconomic status individuals (men and women) from 17 U.S. urban cities, finding that IPV experience was associated with more HIV testing, sex for money or drugs, and unprotected sex with an HIV-positive partner for both genders.

Moreover, some other studies included men who have sex with men (MSM), and only one study included transgender individuals. In the case of MSM sample studies, IPV is referred to as Same-sex Intimate Partner Violence (SSIPV; Murray and Mobley, 2009; Murray et al., 2007). Rosenthal and Starks (2015) studied 192 individuals in same-sex relationships, finding a significant negative association between SSIPV and sexual satisfaction and communication. Ferlatte et al. (2018) found that among 7,872 gay and bisexual men in Canada, SSIPV was associated with syphilis diagnosis. In a study of 436 men, mostly men who have sex with men, Miltz et al. (2019) reported that lifetime and recent SSIPV victimization were positively associated with chemsex and internalized homophobia. Jiang et al. (2020) found that among 976 Chinese men who have sex with men, SSIPV was associated with high-risk sexual behaviors, psychological distress affecting sexual decision-making, physical injuries affecting sexual function, and barriers to sexual health services, such as STI/HIV testing behavior. Hutchinson et al. (2023) noted that sexual orientation, particularly identifying as bisexual, played a significant role in the relationship between SSIPV and sexual and reproductive health outcomes. In addition, a qualitative study by Storholm et al. (2023) with 23 sexually diverse men who experienced IPV revealed a complex relationship between SSIPV and sexual health, in which power dynamics influenced condom use, with cases of coercion and forced unprotected sex. Also, responses to Pre-exposure Prophylaxis (PrEP) varied, and perspectives on HIV/STIs were diverse, including violence after disclosure and intentional transmission by abusive partners. As mentioned above, the only study that included transgender individuals was carried out by Parsons et al. (2018), who conducted a study with 212 transgender individuals from the New York metropolitan area, finding that IPV was associated with higher rates of transactional sex and HIV risk behaviors. These studies collectively underscore the critical intersection of IPV and negative sexual health outcomes in sexually and gender-diverse individuals, highlighting how experiences of IPV can negatively impact not only sexual wellbeing but also efforts for preventing STIs/HIV.

## 4 Discussion

The present systematic review explored the relationship between intimate partner violence and sexual health over the past decade. Key findings revealed complex interactions between these factors, highlighting the need for a nuanced understanding of IPV's impact on sexual health.

Research on IPV and sexual health has evolved significantly from 2014 to 2024. Early contributions by Hellemans et al. (2015) and Overstreet et al. (2015) laid the groundwork for understanding this relationship, while recent studies by Cha et al. (2023) and Callands et al. (2024) have expanded our knowledge. A study from 2014 that aligned with this systematic review's inclusion criteria was not found. The geographical diversity of the research, spanning all five continents, underscores the global relevance of this issue. Studies conducted in diverse settings, ranging from the United States and Canada to Ghana and China, reflect a variety of cultural and socio-economic contexts. Exploring cultural values related to gender, couple relationships, and sexuality is essential as they shape attitudes toward IPV. Also, the concept of “honor culture”, which consists of self-respect, moral behavior, and social status/respect (Cross et al., 2014), could significantly impact perceptions of IPV in different cultural groups (Vandello and Cohen, 2003; Vandello et al., 2009). Regarding the assessment tools for IPV and sexual health, quantitative methodologies dominated the reviewed studies, with standardized instruments like the Conflict Tactics Scale (CTS; Straus et al., 1996) and the Composite Abuse Scale (CAS; Hegarty et al., 2005) being commonly used. These validated tools provided consistent and reliable measures of IPV across different populations. The proliferation of validated instruments in Spanish for assessing IPV represents an important advancement in the field. Nevertheless, in their comprehensive systematic review of psychometric evidence for Spanish-language IPV measures, Hendershot et al. (2024) advocate for the utilization of the Plazaola-Castaño translation of the Spouse Abuse Index (Plazaola-Castaño et al., 2009) when evaluating IPV in Spanish-speaking populations. Within this review, two specific variants of this scale were identified in the analyzed studies: the reduced version by Sierra et al. (2011a) and the version validated for Spanish men by Santos-Iglesias et al. (2013). Both instruments are considered particularly effective measurement tools. The qualitative studies, such as those by Bagwell-Gray (2019) and Storholm et al. (2023), utilized in-depth interviews to capture the nuanced experiences of IPV survivors, offering rich contextual data. These methodologies have enabled a good comprehensive understanding of IPV's impact on sexual health. However, it is important to add that studies using a mixed-methods design could offer a more nuanced insight into the dynamic of IPV and its effects on sexual health. Furthermore, this design would help identify barriers that prevent IPV survivors from seeking help, such as the belief that seeking help is a sign of weakness and the desire to avoid further trauma (Thorvaldsdottir et al., 2022).

Across the studies, IPV was consistently associated with poorer sexual and reproductive health outcomes, especially in heterosexual women. These findings align with those from Coker (2007) and Sierra et al. (2023), both of which show that intimate partner violence (IPV) has harmful effects on women's sexual and reproductive health. Sierra et al. (2023) also found that women who experienced IPV reported lower sexual desire, arousal, and ability to achieve orgasm, along with reduced sexual and relationship satisfaction. The study noted that psychological and physical violence can affect sexual health and overall relationship perception, often leading to dissatisfaction.

Research on cisgender heterosexual men, although limited, revealed similar negative impacts. Moreover, sexually diverse men face unique challenges, with SSIPV linked to high-risk sexual behaviors. It is important to note that research on IPV and sexual health in transgender individuals is very few compared to other groups, highlighting the need for more studies focused on this population. Additionally, no studies addressed the relationship between IPV and sexual health in sexually diverse women or non-binary individuals as their main sample. Conducting research on IPV across diverse population groups is crucial for several reasons: First, studies have demonstrated that IPV prevalence is comparable in heterosexual and same-sex relationships (Signorelli et al., 2012), and a recent study revealed that age structure contributes to higher IPV rates in same-sex couples (Hubbell, 2024). However, IPV in same-sex relationships often goes unreported due to societal homophobia and a lack of appropriate resources (Signorelli et al., 2012). Second, Outlaw et al. (2023) discovered that bisexual individuals face a higher risk of IPV compared to their heterosexual and gay counterparts. Third, Huff et al. (2024) found that nearly half of the transgender and gender-diverse students in their sample reported experiencing some form of IPV victimization. Lastly, as previously mentioned, cultural values related to gender and sexuality significantly influence IPV dynamics. Therefore, considering gender and sexual diversity is essential in addressing IPV and its impact on sexual health. This understanding can guide the development of tailored prevention and support interventions and improve healthcare services to address this public health issue in specific and vulnerable populations.

As previously mentioned, the relationship between IPV and sexual health is complex, aspects such as gender identity, sexual orientation, age, and health status play a direct and significant role in how IPV affects sexual health outcomes. Moreover, aspects such as power dynamics within relationships and communication patterns shape how IPV influences sexual health in a way that affects sexual decision-making, safe sex practices, and overall sexual wellbeing. Contextual factors such as cultural background, socioeconomic status, and geographical location also significantly influence the IPV-sexual health relationship, and, indirectly, such factors can impact sexual health through access to resources, support systems, and healthcare. Research from various studies highlights that lower socioeconomic status is associated with a higher risk of IPV exposure (Larsen, 2016; Gul et al., 2020; Chaurasia et al., 2021), and individuals of lower socioeconomic status often lack the necessary resources to avoid exposure to health risks, including IPV, leading to increased vulnerability (Larsen, 2016).

Overall, understanding these multifaceted influences is crucial for developing effective interventions and policies. A holistic approach that considers the interplay of direct and indirect aspects is necessary to address the complex relationship between IPV and sexual health across diverse populations. The findings of this review have important implications for healthcare providers, counselors, and policymakers. There is a need for targeted interventions that address the specific needs of IPV survivors. Also, future research should aim to be culturally sensitive and consider the intersectionality of these factors to provide a comprehensive understanding for individuals affected by IPV and promote better sexual health outcomes. Healthcare providers should be trained to recognize and respond to IPV-related sexual health issues, while policymakers must develop and implement strategies to protect and support IPV survivors.

### 4.1 Limitations

This systematic review, while comprehensive, has several notable limitations. The predominance of quantitative studies and the scarcity of mixed-methods research may restrict the depth of understanding regarding the complex IPV-sexual health relationship. A significant gap exists in research on transgender individuals –especially transgender men–, sexually diverse women, and non-binary people, limiting our insight into IPV's impact across the full spectrum of gender identities and sexual orientations. The limited research on intimate partner violence (IPV) and its link to sexual health among women in same-sex relationships, particularly lesbian and bisexual women, is shaped by several interrelated factors. One major issue is the historical emphasis on heterosexual women in IPV studies, which has left the experiences of lesbian and bisexual women underrepresented and insufficiently understood (Islam, 2021; Porsch et al., 2023). This gap may be due to challenges in interpreting traditional gender roles, where women are stereotypically seen as “nurturing” and “subordinate” (Stevens et al., 2010). This heteronormative perspective not only oversimplifies IPV dynamics in same-sex relationships but also diminishes the perceived importance of these experiences. Moreover, lesbian and bisexual women often face dual layers of stigma—both societal discrimination based on gender and sexual orientation and biases in healthcare settings as IPV survivors (Islam, 2021; Lo, 2023). The intersecting stigmas faced by queer women reduce the visibility of IPV in their lives, perpetuating the marginalization of their experiences and limiting research on this critical issue (Islam, 2021). Therefore, it is crucial to recognize and address their unique experiences with intimate partner violence, emphasizing the need for a more inclusive and comprehensive approach in both education and support (Harden et al., 2022).

Another key limitation is the geographical bias in the reviewed studies, with an overrepresentation of research from the U.S. and other Western countries. This imbalance may limit the generalizability of findings to diverse cultural and non-Western contexts, where IPV dynamics and health infrastructure differ. Moreover, publication bias favors studies from resource-rich, Western settings, potentially skewing the evidence base. Future research should include more studies from varied global contexts to address this bias. Additionally, there seems to be a limited exploration of intersectionality, potentially overlooking how various social identities interact to influence IPV and sexual health outcomes. Lastly, there appears to be a limited exploration of protective factors and resilience in the face of IPV. These limitations underscore the need for more diverse, comprehensive, and culturally sensitive approaches in future research on the relationship between IPV and sexual health.

## 5 Conclusion

Based on this systematic review, it is evident that intimate partner violence consistently correlates with poorer sexual health outcomes across diverse populations and geographical contexts, underscoring its global significance as a public health issue. The impact of IPV on sexual health varies across gender identities and sexual orientations, with research focusing primarily on cisgender heterosexual women while revealing unique challenges for sexually diverse men and transgender women. The relationship between IPV and sexual health is multi-layered, influenced by a multitude of factors including individual characteristics, relational dynamics, and broader contextual elements such as cultural background and socioeconomic status. Furthermore, the review highlighted the widespread use of standardized instruments, such as the Conflict Tactics Scale and the Composite Abuse Scale, in quantitative research. It also emphasized the availability of Spanish-language measures, including the Spouse Abuse Index. This reflects the growing number of validated instruments accessible to researchers studying intimate partner violence among Spanish-speaking populations. In contrast, qualitative methods provided rich, contextual insights into the experiences of survivors. However, significant research gaps persist, particularly regarding transgender individuals, sexually diverse women, and non-binary people, as well as a lack of mixed-methods studies. Cultural considerations, play a crucial role in shaping attitudes toward IPV and its impact on sexual health, while socioeconomic factors, like lower socioeconomic status, are associated with a higher risk of IPV exposure, emphasizing the need to address social determinants of health in tackling this issue.

Also, this systematic review offers a valuable contribution to the understanding of the intersections between gender, sexuality, and wellbeing, particularly by highlighting the impact of discrimination based on these factors. By examining the relationship between intimate partner violence (IPV) and sexual health, the review demonstrates how gender and sexual orientation profoundly influence individuals' experiences of violence and the resulting health outcomes. Furthermore, the empirical evidence presented underscores the harmful effects of IPV on sexual health, enriching the discourse on discrimination based on gender and sexual orientation. This highlights the urgent need for culturally sensitive approaches in both research and healthcare, ensuring that the unique needs of diverse populations are addressed. In addition, the findings advocate for the development of policies aimed at protecting and supporting survivors of IPV, ultimately promoting wellbeing across different communities. This review not only deepens our understanding of the complex dynamics involved but also emphasizes the importance of addressing the specific challenges faced by marginalized and vulnerable groups. By doing so, it calls for the creation of a more equitable and supportive environment for all individuals and all forms of romantic relationships.

## Data Availability

The original contributions presented in the study are included in the article/supplementary material, further inquiries can be directed to the corresponding author.
